# Cardiovascular disease risk in patients with hepatitis C infection: Results from two general population health surveys in Canada and the United States (2007-2017)

**DOI:** 10.1371/journal.pone.0208839

**Published:** 2018-12-12

**Authors:** Alaa Badawi, Giancarlo Di Giuseppe, Paul Arora

**Affiliations:** 1 Public Health Risk Sciences Division, Public Health Agency of Canada, Toronto, ON, Canada; 2 Department of Nutritional Sciences, Faculty of Medicine, University of Toronto, Toronto, ON, Canada; 3 Dalla Lana School of Public Health, University of Toronto, Toronto, ON, Canada; 4 Division of Enteric Diseases, National Microbiology Laboratory, Public Health Agency of Canada, Toronto, ON, Canada; Duke University, UNITED STATES

## Abstract

The role of hepatitis C virus (HCV) infection in increasing the risk of cardiovascular disease (CVD) is controversial. The objective of the present study is to estimate the 10-year risk of CVD in HCV- positive subjects and describe their profile of cardiometabolic risk markers compared to HCV-negative subjects. We conducted a cross-sectional study to estimate 10-year CVD risk, calculated using the Framingham Risk Score (FRS), in participants from the Canadian Health Measures Survey (CHMS; 2007–2015, *n* = 10,115) and the US-National Health and Nutrition Examination Survey (NHANES; 2007–2016, *n* = 16,668). Subjects included in our analysis were aged 30 to 74 years with no prior history of CVD. FRS estimates, sociodemographic and cardiometabolic risk factors were compared between HCV- positive and -negative subjects in the two surveys. HCV-positive subjects had a distinct sociodemographic profile compared to their HCV-negative counterparts. Cardiometabolic risk factors, inflammatory markers and serum levels of micronutrients were comparable between the two survey populations, both in HCV-positive and -negative subjects. The average FRS in HCV-positive patients was in the range of “intermediate” 10-year CVD risk (*i*.*e*., 10–20%) and was significantly higher (*P*<0.01) than their HCV-negative counterparts who were within the “low” 10-year CVD risk range (*i*.*e*., ≤10%). Using a multivariable linear regression model adjusted for ethnicity, number of metabolic syndrome components and BMI, HCV infection was significantly associated with a 2.5–3.5% absolute risk increase of 10-year CVD (*P*<0.01). The results of the present study suggest a potential association between HCV infection and risk of subclinical and clinical CVD. The expansion of anti-HCV therapy may also contribute to reduced CVD risk and burden in patients with chronic HCV infection and should be explored further in other datasets and population modelling studies.

## Introduction

HCV infection is endemic worldwide, with an estimated global prevalence of 2.5–3%, causing chronic liver diseases in 170–200 million people [1–3]. Presently, HCV is the leading cause of progressive liver fibrosis, resulting in cirrhosis, liver cancer, liver failure and death [2–4]. The rate of mortality amongst persons living with HCV currently surpasses that of those living with human immunodeficiency virus (HIV) infections [5–6]. HCV exerts its main effects in the liver leading to hepatic fibrosis and subsequent cirrhosis in about 20% of the chronically infected cases [7]. HCV infection may also exert an effect extrahepatically [8, 9] leading to abnormal endocrine, hematologic, neurologic and renal functions [10, 11]. In addition to liver diseases, over the past decade, several longitudinal studies have shown that patients with chronic HCV infection also experience increased rates of cause-specific mortality from cardiovascular disease (CVD) [12–16].

The association of HCV infection with CVD is still an issue of controversy. Some reports have demonstrated a higher CVD risk among HCV-infected persons [17, 18] whereas others have not reported such an association [19, 20]. The role of HCV in the development of CVD was thought to be related to interference of the infection with glucose and lipid metabolism [21] that may result in the development of metabolic syndrome and subsequent risk of developing insulin resistance (IR), type 2 diabetes and CVD. Beyond this metabolic dysregulation, HCV infection was also thought to increase CVD risk via mechanisms that facilitate chronic inflammation and/or endothelial dysfunction [21].

With CVD presently being the leading cause of death worldwide [22], an improved understanding of disease risk may help tailor interventions and prevention efforts in patients infected with HCV. A number of prediction scores such as the Framingham Risk Score (FRS) [23], European Systematic Coronary Risk Evaluation (SCORE) function [24], the PROCAM (Prospective Cardiovascular Münster) model [25], the QRISK function [26] and the Reynolds functions [27, 28] have been developed to estimate 10-year risk of cardiovascular outcomes. These prediction models are used to identify high-risk patients who would benefit from interventions on one or more risk factors. The FRS is the most commonly used risk prediction score in North America [23] both for clinical and research purposes [23, 29]. It was developed in the USA [23] and validated in Canada [29] as a means to predict CVD risk in asymptomatic patients. This gender-specific multivariate function (algorithm) estimates risk of CVD including coronary heart diseases, cerebrovascular events, peripheral artery disease and heart failure [23, 30].

There are several distinct FRS models which can be used for either diabetic or non-diabetic patients. In general, FRS combines the information in CVD risk factors such as sex, age (usually, 30–74 years), systolic blood pressure, total cholesterol (T-Chol), high-density lipoprotein cholesterol (HDL-C), smoking behavior, the status of diabetes and use of antihypertensive medications to produce the 10-years estimate (or risk) of developing CVD. CVD events estimated from the FRS are defined as coronary death, myocardial infarction, coronary insufficiency, angina, ischemic stroke, hemorrhagic stroke, transient ischemic attack, peripheral artery disease, and health failure [23]. These estimates of CVD risk can subsequently be used to guide pharmacological or behaviour-based preventive measures.

The objective of the present study is to determine the association between 10-year risk of CVD and HCV infection status in otherwise healthy subjects from nationally representative surveys, namely the Canadian Health Measures Survey (CHMS) and the National Health and Nutrition Examination Survey (NHANES) from Canada and USA, respectively. This measure will provide additional information to encourage further debate on the potential role of HCV in CVD risk, and ultimately contribute to the body of evidence needed to explore the development of more targeted CVD preventive actions for those living with HCV.

## Methods

### Study population and eligibility criteria

Data were collected from the Canadian Health Measures Survey (CHMS) and the US National Health and Nutrition Examination Survey (NHANES). Both CHMS and NHANES are cross-sectional surveys of the noninstitutionalized civilian Canadian and US resident populations, respectively, designed to collect information on the health and wellness as well as nutrition status of both populations.

The CHMS survey is conducted by Statistics Canada to collect information from Canadians aged 3 to 79 years, from households in the ten provinces and three territories of Canada. Those living on Aboriginal Reserves or Crown Lands, in institutions and certain remote regions, and full-time members of the Canadian Forces were not captured by this survey. The CHMS covers approximately 96.3% of the Canadian population at the selected age group. CHMS data included in this study were obtained from cycles 1 to 4 collected between March 2007 and December 2015 (Cycle 1: March 2007 to February 2009, n = 5,604; Cycle 2: August 2009 to November 2011, n = 6,395; Cycle 3: January 2012 to December 2013, n = 5,785 and Cycle 4: January 2014 to December 2015, n = 5,794). Comprehensive details of the CHMS multistage sampling design and data collection methods have been previously published [31–33]. Briefly, a multi-stage sampling strategy was used to identify the sites from which data was collected. Participants (n = 23,578) signed a consent form prior to participating in the study. Participation was voluntary and included a household interview that included general sociodemographic questions as well as an in-depth health questionnaire. Subsequently, participants visited a mobile examination centre (MEC) where physical and biological measurements were taken. The study was reviewed and approved by the Health Canada Research Ethics Board [34].

The NHANES survey is conducted by the National Center for Health Statistics (NCHS), CDC. The survey examines a nationally representative sample of approximately 5,000 individuals of all age groups each year from all counties across the USA. All of the study methods were approved by the NCHS research ethics review board. All of the participants provided informed consent. NHANES participants are selected by using a complex multistage sampling design [35]. Similar to the CHMS, the NHANES includes an in-home health interview and a physical examination in a MEC in addition to a follow-up telephone interview. This analysis includes data from five cycles of NHANES 2007/08, 2009/10, 2011/12, 2013/14, and 2015/16 (n = 50,526). Detailed methods of the NHANES survey construction and sampling strategy have been previously described [36, 37]. Each NHANES survey cycle is a stratified, multistage, probability random sample designed to represent the noninstitutionalized house-dwelling US civilian population.

**Fig 1:**
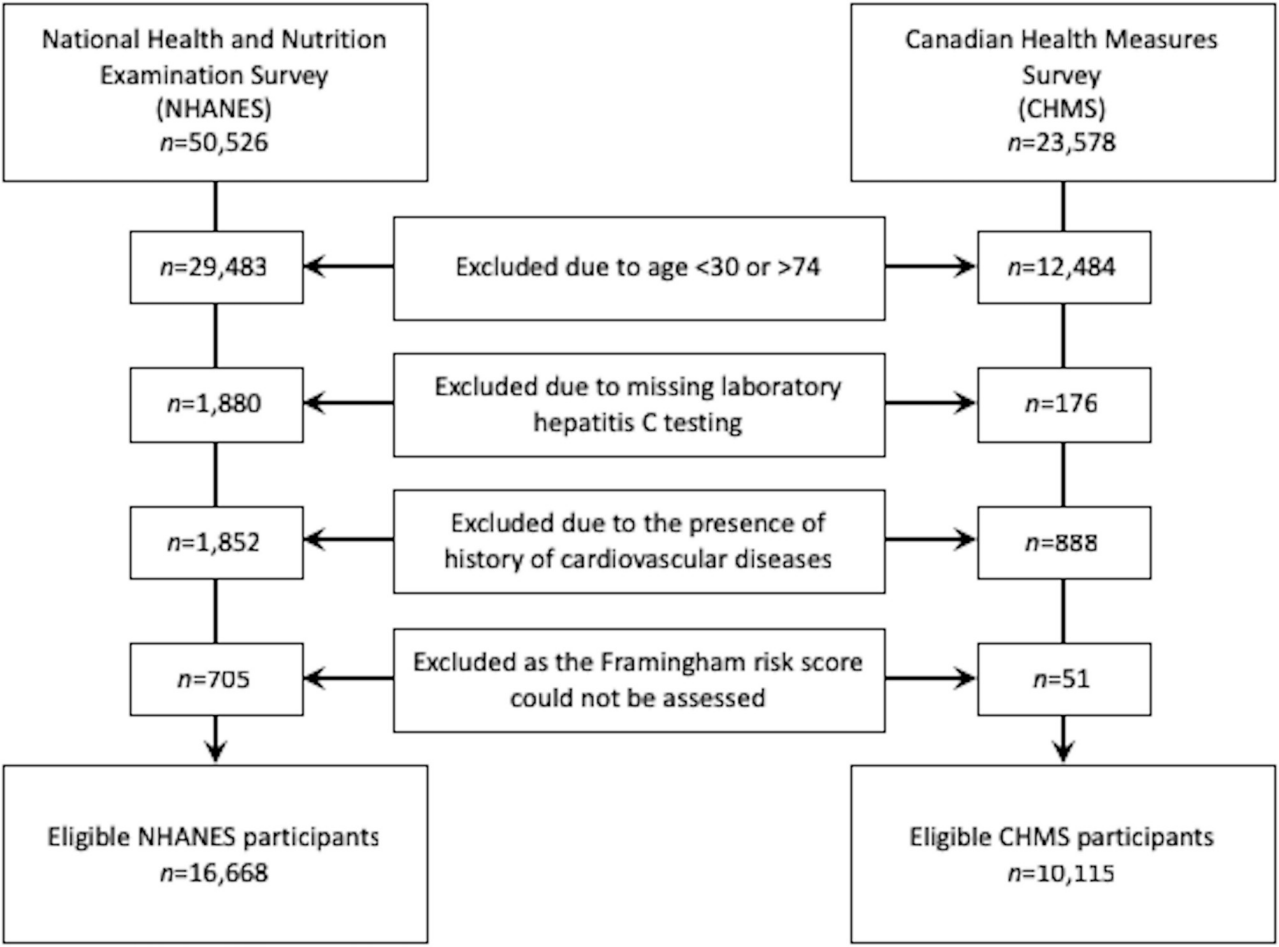
Flowchart of study population selection.

The FRS algorithm was developed in a study population aged 30 to 74 without a history of CVD event at baseline. Therefore, we excluded subjects under the age of 30 years or over the age of 74 years (CHMS: n = 12,348; NHANES: n = 29,483) as they were not relevant to the FRS algorithm. FRS guidelines restricts the use this tool for people between 30 and 74 years old [23]. History of CVD was assessed via self-report. Survey participants were asked by health professionals if they have or had heart disease and they were asked if they have ever been told by health professional that they have or had heart attack. An answer of “yes” to any of these questions resulted in exclusion from the current study. Patients with confirmed (or with a history of) heart diseases (CHMS: n = 888; NHANES: n = 1,852) were excluded. Participants with missing data for any of the components used in the algorithm of the FRS (i.e. age, gender, blood pressure, T-Chol, HDL-C, smoking status, diabetes, anti-hypertensive medication) were also excluded as the FRS could not be calculated (CHMS: n = 51; NHANES: n = 705). Moreover, participants missing information on HCV status or with an indeterminate HCV infection (CHMS: n = 176; NHANES: n = 1,880) were not included. Ultimately, the total number of participants included in the present study was 10,115 subjects from the CHMS survey (male:female ratio of 1:1.12) and 16,668 subjects (male:female ratio of 1:1.09) from the NHANES survey (see Fig 1). This group was further divided to HCV+ (CHMS: n = 87; NHANES: n = 326) and HCV- (CHMS: n = 10028; NHANES: n = 16,342) subgroups.

### Hepatitis C infection and cardiovascular disease risk

In both surveys, infection with HCV was classified using laboratory-confirmed samples. Infection status was determined using serum specimens screened for anti-HCV antibodies or HCV RNA. A participant was considered “HCV-positive” if a confirmed antibody test yielded positive results or viral HCV RNA was present in the serum. Participants with an indeterminate antibody test and negative RNA test were considered “HCV-negative”, whereas those with indeterminate anti-HCV and without RNA testing were considered “unknown” and were excluded as HCV status could not be determined with certainty. HCV infection was detected using VITROS Anti-HCV Assay (Ortho-Clinical Diagnostics, Raritan, NJ, USA) in both surveys. All samples positive on initial screening for anti-HCV were confirmed by INNO-LIA HCV Score immunoblot assay (Innogenetics, Fugirebio Inc., GA, US) in the CHMS, and by Chiron RIBA 3.0 Strip Immunoblot Assay (SIA; Chiron Corporation, Inc.) in the NHANES. Indeterminant and positive HCV samples in NHANES were further tested for viral RNA in the serum samples using the COBAS AMPLICOR HCV Test, version 2.0 (Roche Diagnostics Corp., Indianapolis, IN). In the NHANES HCV-positive individuals (*n* = 326), a total of 239 had anti-HCV test results and 304 had HCV-RNA testing done. HCV status was assigned by only one lab test result using anti-HCV and HCV-RNA only in 22 and 87 individuals, respectively. A total of 217 had both anti-HCV and HCV-RNA testing done with 167/217 had both a positive anti-HCV and HCV-RNA test, and 48/217 had a positive anti-HCV test but negative HCV-RNA test, while the remaining 2 having indeterminant anti-HCV test but positive HCV-RNA results (S1 Table). All 87 CHMS HCV-positive patients had anti-HCV testing performed. Among those, 21 subjects were also examined for HCV-RNA, 14 of which were positive for HCV-RNA. In participants from both surveys, information on the use of the recent direct-acting antivirals were not available.

### Estimating the 10-year risk of CVD

Estimates of 10-year CVD risk in asymptomatic subjects was determined using the FRS points-based method [23] in HCV positive (HCV+) and HCV-negative (HCV-) subjects. The FRS algorithm has been described extensively previously [23] and has been validated in the USA [23] and Canada [29], both in White and African American subjects. We calculated the raw FRS score for each participant based on the individual's sex, age, systolic blood pressure, T-Chol, HDL-C, smoking status (smoker or non-smoker), presence or absence of diabetes and the use of antihypertensive medications. The points assigned for each risk factor are based on the value for the β-coefficient of the proportional hazard regressions [23, 29]. The 10-year risk score is then derived as a percentage. As previously described [29], 10-year CVD risk is considered low if the FRS-derived estimate is less than 10%, moderate if it is 10% to 19%, and high if it is 20% or higher.

### Sociodemographic factors, metabolic markers and other covariates

Sociodemographic information was captured through responses to questionnaires given during the structured interview portion of each survey and included: age, gender, ethnicity, education, marital status, history of injection drug use, and household income. Ethnicity was categorized into 4 main subgroups: White, African Americans, Asian (i.e., Korean, Filipino, Japanese, Chinese, South Asian, Southeast Asian, Arab, and West Asian), and Other (i.e. Latin American or mixed racial origins). Household income was dichotomized according to self-reported income below or above $20,000 per annum. Self-reported smoking status was categorized into smokers (daily/occasional) and non-smokers.

A number of metabolic markers were measured in both the CHMS and NHANES and were available for the present study. These included cardiometabolic disease markers (apolipoprotein [Apo] A1 [g/L], ApoB [g/L], LDL-C [mmol/L], HDL-C [mmol/L], T-Chol [mmol/L], T-Chol:HDL-C ratio, triglycerides [mmol/L], and glycosylated hemoglobin [HbA1c] [%]); inflammatory biomarkers (CRP [mg/L], fibrinogen [g/L], and homocysteine [μmol/L]); systolic and diastolic blood pressure; and plasma 25-hydroxyvitamin D (25(OH)D) (nmol/L) [38]. Factors related to obesity such as body mass index (kg/m^2^), waist circumference (cm) and waist-to-hip were all assessed as previously described [38]. Diabetes status was defined as a self-reported or HbA1c ≥ 6.5% [39]. The US National Cholesterol Education Program Adult Treatment Panel III (ATPIII) criteria were used to define metabolic syndrome [40, 41]. The presence of at least three of the following five metabolic risk factors constitutes a diagnosis of metabolic syndrome: abdominal obesity (waist circumference ≥102 cm in men and ≥88 cm in women), elevated triglyceride level (1.7 mmol/L), reduced HDL-C level (1.0 mmol/L in males; 1.3 mmol/L in females), elevated blood pressure (systolic ≥ 130 and/or diastolic ≥ 85 mmHg), and elevated fasting plasma glucose level (≥5.6 mmol/L) [41]. Individuals who have already been diagnosed as hypertensive, diabetic, or those who were using antihypertensive drugs were included [40–42]. Insulin resistance (IR) was approximated using the homeostatic model assessment (HOMA-IR) formula [(glucose (mmol/L) x insulin (μIU/mL)) ÷ 22.5] [43, 44]. Liver functions were evaluated in both survey studies using serum enzyme markers of alanine aminotransferase, alkaline phosphatase, aspartate aminotransferase, lactate dehydrogenase and γ-glutamyl transferase. Furthermore, A number of serum micronutrients including vitamins B12, C, and D were measured in both the CHMS and NHANES and were captured in the present study.

### Statistical analysis

All analyses were stratified by HCV infection status and by RNA positivity within the HCV positive cases and performed separately on Canadian and US participants. Guidelines and principles published by NHANES and CHMS were used to combine survey data over multiple cycles [23, 45, 46]. Data from each cycle were treated as a completely random population sample and survey weights were excluded from all analyses. Frequency distributions and means (±standard deviation) were used to describe baseline characteristics. Associations between HCV group and baseline characteristics were determined using *t*-test and *χ*^*2*^ tests for continuous and categorical variables, respectively. Fisher’s exact test was used for categorical data analysis where there were small sample sizes. Sociodemographic characteristics and levels of biomarkers and cardiometabolic risk factors as well as the FRS values were all compared between HCV-positive and negative cases as well as between RNA-positive and—negative cases in HCV infected subjects. Linear regression was used to explore the association between FRS (outcome = 10-year CVD risk) and HCV infection status. Unadjusted and adjusted regression analyses were conducted with two models hypothesized *a priori*. A multivariable regression model was used to control for ethnicity, BMI, and the number of Adult Treatment Panel III (ATPIII) components [47]. We used the ATPIII score in the multivariable model since it is a composite measure of factors accounting for overall cardiovascular health. The degree of missing data was assessed for each variable and was considered for multivariable regression model inclusion. If a variable had >80% missing data, it wouldn’t be fit to be included in the regression model. There was no statistically significant difference between the proportion of missing data and HCV status, thus data was considered missing at random. All analyses were conducted using SAS version 9.4 (SAS Institute, Cary, North Carolina) at the Public Health Agency of Canada.

## Results

A total of 23,578 Canadians responded to the CHMS during the study period, of which 10,115 (43%) were eligible for this study. Similarly, a total of 50,588 individuals in the USA responded to five cycles of NHANES, and 16,668 (33%) met eligibility criteria. The selection procedure of the study population is illustrated in Fig 1. The prevalence of HCV among NHANES participants (1.95%, *n* = 326) was about 2-fold higher than their CHMS counterparts (0.9%, *n* = 87).

Baseline sociodemographic characteristics of the study population stratified by HCV status are shown in Table 1 for each survey. Although the Canadian population was predominantly Whites (80%), there was higher ethnic diversity in the NHANES survey. Except for gender frequency, and use of hypertensive medications in the CHMS study, all sociodemographic characteristics were significantly different between the HCV-infected patients and non-infected subjects. In both surveys, HCV-infected individuals were, on average, older, had higher frequency of being single, lower education level, lower income, and with increased smoking rates compared to their non-infected counterparts (see Table 1). Recreational (intravenous) drug use was significantly (*P*<0.001) higher in HCV-infected patients than non-infected subjects both in the USA (33% vs. 1%) and Canada (53% vs. 1%). As shown in Table 2, when HCV positive cases in each survey were stratified by the RNA positivity (i.e., the status of viremia), no significant differences were shown in the Canadian or the US subjects between HCV-RNA-negative and -positive cases. Exceptions in the NHANES HCV-positive cases included a significant age increase and higher rates of using antihypertensive medications in the HCV-RNA-positive cases.

**Table 1:** Sociodemographic characteristics of the study population stratified by the hepatitis C virus infection.

Characteristic		CHMS					NHANES				
		HCV -		HCV +			HCV-		HCV +		
		*n*	(%)^2^	*n*	(%)	*P*^3^	*n*	(%)	*n*	(%)	*P*^3^
**Respondents**		10028	100	87	100		16342	100	326	100	
**Males**		4722	47	46	53		7753	47	212	65	<0.001
**Age (yr), (mean±SD)**^**1**^		49**±**13		51**±**10		0.033	50**±**12		53**±**9		<0.001
**Ethnicity**											
	White	8070	80	70	80	0.002	3265	20	104	32	<0.001
	African Americans	245	2	2	2		4650	28	73	22	
	Asian	1138	11	3	3		1274	8	4	1	
	Other	575	6	12	14		7153	44	145	44	
**Marital Status**											
	Married	7076	71	30	34	<0.001	10885	67	161	49	<0.001
	Divorced	1729	17	24	28		3504	21	114	35	
	Single	1223	12	33	38		1953	12	51	16	
**Highest Level of Education Obtained**											
	Less than grade 12	1111	11	20	23	**<0.001**	4046	25	125	38	<0.001
	High-school graduate	1954	19	28	32		8084	49	180	55	
	Post-secondary graduate	6859	68	39	45		4203	26	20	6	
**Household Income**											
	< $20,000 per annum	729	7	36	41	**<0.001**	2824	17	125	38	<0.001
**History of intravenous drug use**		85	1	46	53	**<0.001**	147	1	108	33	<0.001
**Metabolic syndrome, n (%)**		10028		87			16342		326		
<3		8644	86	76	87		10611	65	218	67	
≥3		1384	14	11	13		5731	35	108	33	
**Medication Use for Hypertension**		1677	17	16	18		4187	25	104	30	0.016
**Diabetes, n (%)**^**4**^		789	8	11	13		2313	14	46	14	
**Smoking Status**											
	Daily / Occasional	1832	18	57	66	**<0.001**	3767	23	199	61	<0.001
	Non-smoker	8196	82	30	34		12575	77	127	39	

^**1**^Only respondents aged 30–74 years were considered for the purpose of this study.

^**2**^Unweighted frequency.

^**3**^χ^2^ test for the difference between hepatitis C virus-positive (HCV+) and -negative (HCV-) cases. Only significant differences are shown.

^**4**^Diabetes defined as Hb1Ac ≥ 6.5% or self-reported diabetic as per survey questionnaire.

**Table 2:** Sociodemographic characteristics of the hepatitis C virus (HCV) infected subjects stratified by the HCV-RNA status.

Characteristic		CHMS (*n* = 87)					NHANES (*n* = 326)			
		HCV-RNA-		HCV-RNA+			HCV-RNA-		HCV-RNA+		
		*n*	%^2^	*n*	%	*P*^3^	*n*	(%)	*n*	(%)	*P*^3^
**Respondents (underwent RNA analysis)**											
Yes (*n*)		7		14			48		256		
No (*n*)		66					22				
**Males**		3	43	9	64		35	73	165	64	
**Age (yr), (mean±SD)**^**1**^		52**±**13		53**±**9			50**±**9		53**±**9		0.039
**Ethnicity**											
	White	6	86	10	72		7	15	92	36	0.021
	African Americans			1	7		12	25	55	22	
	Asian	1	14						3	1	
	Other			3	21		29	60	106	41	
**Marital Status**											
	Married	3	43	4	29		26	54	123	48	
	Divorced	2	29	4	29		13	27	94	37	
	Single	2	29	6	42		9	19	39	15	
**Highest Level of Education Obtained**											
	Less than grade 12			5	36		14	29	103	40	
	High-school graduate	3	43	4	28		28	58	138	54	
	Post-secondary graduate	4	57	5	36		6	13	14	6	
**Household Income**											
	< $20,000 per annum	2	29	7	50		15	33	103	40	
**History of intravenous drug use**		2	29	7	50		20	43	81	32	
**Metabolic syndrome, n (%)**		7		14			48		256		
** **<3		5	71	13	93		32	67	172	67	
** **≥3		2	29	1	7		16	33	84	33	
**Medication Use for Hypertension**		1	14	2	14		8	17	83	32	0.029
**Diabetes, n (%)**^**4**^		1	14	3	21		3	6	39	15	
**Smoking Status**											
	Daily / Occasional	3	43	10	71		24	50	163	64	
	Non-smoker	4	57	4	29		24	50	93	36	

^**1**^Only respondents aged 30–74 years were considered for the purpose of this study.

^**2**^Unweighted frequency within the corresponding HCV-RNA status.

^**3**^χ^2^ test for the difference between hepatitis C virus-RNA-positive (HCV-RNA+) and -negative (HCV-RNA-) cases. Only significant differences are shown.

^**4**^Diabetes defined as Hb1Ac ≥ 6.5% or self-reported diabetic as per survey questionnaire.

Empty cells denote no subjects are available in the corresponding characteristic.

Mean levels of cardiometabolic biomarkers, factors related to obesity, inflammatory markers, micronutrients and liver enzyme markers are presented in Table 3 and stratified by the HCV infection status. The mean levels of these factors were all within normal clinical ranges regardless of HCV infection status. However, serum levels of triglycerides, LDL-C, T-Chol:HDL-C, and ApoB were all significantly lower in HCV-positive participants than in non-infected subjects. The HCV-positive participants from the CHMS had higher total cholesterol than non-infected Canadians (4.50±0.97 vs. 3.94±1.27 mmol/L; *P*<0.001). In contrast, HCV-positive participants from the NHANES had lower total cholesterol than the non-infected Americans (4.67±1.0 vs. 5.17±1.1 mmol/L; *P*<0.001). HCV status was associated with a lower body mass index in both survey populations. There was no association between the diabetic status (self-reported or using the Hb1Ac cut-off ≥6.5%) and HCV-infection status. Similarly, the frequency of metabolic syndrome did not differ between HCV-infected and non-infected subjects both in the CHMS and the NHANES surveys. Levels of inflammatory markers and concentrations of micronutrients were similar in both infected and control subjects with the exception of CRP (in NHANES) and fibrinogen (in CHMS) that were slightly—but significantly—lowered in the HCV-infected patients. As expected, all serum markers for liver functioning enzymes were significantly elevated in HCV-positive subjects. As shown in Table 4, when HCV positive cases in each survey were stratified by the RNA positivity, although from a small sample size, there was a tendency—between the two surveys HCV-RNA-positive cases—to have higher levels of systolic blood pressure and lower total cholesterol, LDL-C, total cholesterol:HDL-C ratio and ApoB than the RNA-negative HCV infected cases. As expected during the active HCV infection, liver enzymes were significantly increased in the HCV-RNA-positive cases compared to the -negative ones. Furthermore, FRS value, only in the NHANES HCV-positive cases, was significantly (*P* = 0.047) higher in the RNA-positive cases (15.3±10.4%) than in the -negative (12.1±9.8%) cases.

**Table 3:** Levels of biomarkers and cardiometabolic risk factors in the study populations stratified by the hepatitis C virus infection.

Biomarker		CHMS					NHANES				
		HCV - (*n* = 10028)		HCV + (*n* = 87)		*P*^1^	HCV - (*n* = 16342)		HCV + (*n* = 326)		*P*^1^
		*n*	mean±SD	*n*	mean±SD		*n*	mean±SD	*n*	mean±SD	
**Cardiometabolic risk markers**											
	Systolic blood pressure (mmHg)	10028	114**±**15	87	115**±**21		16342	123**±**17	326	128**±**21	<0.001
	Diastolic blood pressure (mmHg)	10028	73**±**9	87	75**±**10	0.031	16342	72**±**12	326	74**±**14	0.013
	Triglycerides, fasting (mmol/L)	5066	1.43**±**0.89	41	1.15**±**0.52	0.047	6412	1.52**±**1.4	132	1.32**±**0.9	0.015
	Total cholesterol (mmol/L)	10028	3.94**±**1.27	87	4.50**±**0.97	<0.001	16342	5.17**±**1.1	326	4.67**±**1.0	<0.001
	LDL-C (mmol/L)	5023	3.03**±**0.92	41	2.48**±**0.89	<0.001	6269	3.09**±**0.9	130	2.69**±**0.9	<0.001
	HDL-C (mmol/L)	10028	1.38**±**0.40	87	1.42**±**0.45		16342	1.37**±**0.4	326	1.36**±**0.5	
	Total cholesterol:HDL-C ratio	10028	3.94**±**1.27	87	3.37**±**1.01	<0.001	16342	4.10**±**1.5	326	3.84**±**1.9	0.013
	Insulin, fasting (pmol/L)	5016	77**±**67	41	94**±**62		4715	83**±**95	104	102**±**119	
	Glucose, fasting (mmol/L)	2418	5.30**±**1.26	23	5.63**±**2.23		6402	6.05**±**2.0	132	6.28**±**2.2	
	HOMA-IR	2417	3.5**±**3.9	23	4.4**±**3.5		4706	4.0**±**5.6	104	5.4**±**11.3	
	HbA1c (%)	9774	5.61**±**0.77	85	5.67**±**0.88		16313	5.8**±**1.1	326	5.7**±**1.1	
	Apolipoprotein A1, fasting (g/L)	3661	1.46**±**0.27	32	1.49**±**0.28		*–*^**2**^	*–*	*–*	*–*	
	Apolipoprotein B, fasting (g/L)	3660	0.97**±**0.26	32	0.75**±**0.20	<0.001	6412	0.95**±**0.2	132	0.85**±**0.2	<0.001
**Obesity**											
	Body mass index (kg/m^2^)	10004	27.9**±**5.7	85	26.9**±**5.7		16219	29.5**±**6.8	321	28.1**±**6.5	<0.001
	Waist circumference (cm)	9927	94.6**±**15.3	87	94.5**±**15.7		15884	100.1**±**15.7	313	98.4**±**15.2	
	Waist-to-hip ratio	9918	0.90**±**0.1	86	0.93**±**0.1	0.004	*–*	*–*	*–*	*–*	
**Inflammatory markers**											
	C-reactive protein (mg/L)	9754	2.5**±**2.9	84	2.5**±**3.2		6750	4.2**±**7.8	171	3.1**±**6.2	0.026
	Fibrinogen (mmol/L)	5190	3.1**±**0.6	39	2.9**±**0.6	0.042	*–*	*–*	*–*	*–*	
	Homocysteine (umol/L)	2524	8.2**±**3.0	18	9.0**±**3.3		*–*	*–*	*–*	*–*	
**Micronutrients**											
	Vitamin B12 (pmol/L)	9804	340**±**193	85	362**±**145		6396	462**±**434	115	462**±**209	
	Vitamin D (nmol/L)	9985	64**±**26	87	64**±**27		9404	63**±**26	147	63**±**31	
	Vitamin C (nmol/L)	1226	52**±**23	4	33**±**43		*–*	*–*	*–*	*–*	
**Liver enzyme markers**											
	Alanine aminotransferase (U/L)	7243	33**±**16	61	63**±**54	<0.001	16284	26**±**19	325	57**±**44	<0.001
	Alkaline phosphatase (U/L)	9985	78**±**24	86	90**±**36	0.004	16288	69**±**24	325	77**±**29	<0.001
	Aspartate aminotransferase (U/L)	9984	28**±**12	86	55**±**47	<0.001	16282	26**±**18	325	57**±**43	<0.001
	γ-Glutamyl transferase (U/L)	10000	32**±**37	86	92**±**176	0.002	16289	30**±**43	325	80**±**103	<0.001
	Lactate dehydrogenase (U/L)	2291	381**±**86	17	427**±**67	0.028	16281	129**±**31	325	142**±**37	<0.001

^**1**^t-test for the difference between hepatitis C virus positive (HCV+) and negative (HCV-) case. Only significant differences are shown.

^**2**^Variable are not captured by survey.

**Table 4:** Levels of biomarkers and cardiometabolic risk factors in the hepatitis C virus (HCV) infected subjects stratified by the HCV-.

Biomarker		CHMS (*n* = 87)					NHANES (*n* = 326)				
		HCV-RNA- (*n* = 7)		HCV-RNA+ (*n* = 14)		*P*^1^	HCV-RNA- (*n* = 48)		HCV-RNA+ (*n* = 256)		*P*^1^
		*n*	mean±SD	*n*	mean±SD		*n*	mean±SD	*n*	mean±SD	
**Cardiometabolic risk markers**											
	Systolic blood pressure (mmHg)	7	119**±**16	14	130**±**36		48	122**±**18	256	129**±**21	0.03
	Diastolic blood pressure (mmHg)	7	78±10	14	79±11		48	73±10	256	74±15	
	Triglycerides, fasting (mmol/L)			4	1.16±0.67		16	1.31±0.48	101	1.35±1.01	
	Total cholesterol (mmol/L)	7	4.5±1.2	14	4.24±0.64		48	5.06±1.13	256	4.60±0.98	0.011
	LDL-C (mmol/L)			4	1.68±0.47		16	3.51±1.05	99	2.56±0.83	0.003
	HDL-C (mmol/L)	7	1.24±0.45	14	1.70±0.68		48	1.31±0.43	256	1.38±0.54	
	Total cholesterol:HDL-C ratio	7	3.89±1.02	14	2.74±0.74	0.026	48	4.27±1.75	256	3.77±1.95	
	Insulin, fasting (pmol/L)			4	88±37		16	97±86	74	105±133	
	Glucose, fasting (mmol/L)			4	7.95±5.02		16	6.10±1.87	101	6.35±2.40	
	HOMA-IR			4	6.1±6.0		16	4.3±3.8	74	5.9±13.3	
	HbA1c (%)	7	5.56±1.07	13	5.97±1.58		48	5.52±0.79	256	5.77±1.18	
	Apolipoprotein A1, fasting (g/L)			4	1.67±0.41						
	Apolipoprotein B, fasting (g/L)			4	0.73±0.20		16	1.06±0.27	101	0.82±0.23	0.003
**Obesity**											
	Body mass index (kg/m^2^)	7	27.01±5.42	14	23.75±4.05		47	27.38±5.67	253	28.14±6.41	
	Waist circumference (cm)	7	98.2±13.1	14	89.1±14.1		47	97.9±14.3	246	98.3±15.4	
	Waist-to-hip ratio	7	0.95±0.08	14	0.93±0.1						
**Inflammatory markers**											
	C-reactive protein (mg/L)	6	4.27±4.01	13	0.93±0.92		35	5.01±11.26	122	2.75±4.03	
	Fibrinogen (mmol/L)										
	Homocysteine (umol/L)										
**Micronutrients**											
	Vitamin B12 (pmol/L)	7	371±229	14	355±169		13	452±210	95	459±188	
	Vitamin D (nmol/L)	7	53±25	14	68±39		33	68±33	98	61±31.3	
	Vitamin C (nmol/L)			4	33±43						
**Liver enzyme markers**											
	Alanine aminotransferase (U/L)						48	26±18	255	64±45	<0.001
	Alkaline phosphatase (U/L)	7	99±46	14	96±31		48	71±23	255	79±30	
	Aspartate aminotransferase (U/L)	7	35±19	14	68±46	0.029	48	31±26	255	62±43	<0.001
	γ-Glutamyl transferase (U/L)	7	103±185	14	142±228		48	33±29	255	90±112	<0.001
	Lactate dehydrogenase (U/L)						48	138±57	255	143±32	
**FRS (%)**		7	11.0±12.5	14	13.5±8.3		48	12.1±9.8	256	15.3±10.4	0.047

RNA status.

^**1**^t-test for the difference between hepatitis C virus-RNA-positive (HCV-RNA+) and -negative (HCV-RNA-) cases. Only significant differences are shown.

Empty cells denote either variables that were not captured by survey (see Table 3) or no subjects are available in the corresponding biomarker.

As shown in Table 5, the average 10-year risk of CVD estimated by the FRS (%) was higher in the HCV-infected group when compared to uninfected participants from the USA (14.7±10.3 vs. 10.0±10.0; *P*<0.001) and Canada (10.5±8.8 vs. 8.0±6.6; *P* = 0.008). Furthermore, the CVD risk was found to be higher among NHANES participants on a population level when compared to Canadians in both HCV-infected (*P*<0.0001) and uninfected (*P* = 0.0006) subjects. There was no difference between HCV-RNA status in the mean FRS score between HCV-RNA positive (14.14 ± 10.03%) and RNA-negative (12.06 ± 9.76) individuals who were positive for anti-HCV antibodies (*P* = 0.20) from the NHANES population. Furthermore, in the CHMS study, there was no significant difference in the mean FRS score between the RNA-HCV-positive (13.46±8.65) and -negative subjects (11.03±12.54) among those 21 individuals who had both antibody and RNA testing (*P* = 0.60). In an unadjusted linear regression model, HCV infection was associated with a 4.21% (*P*<0.001) and 2.47% (*P* = 0.008) higher 10-year CVD risk estimated by the FRS in the American and Canadian participants, respectively. After controlling for ethnicity, number of metabolic syndrome components, and body mass index, HCV infection remained associated with a higher CVD risk. The difference between HCV-positive and HCV-negative subjects in 10-year absolute risk of CVD was higher in American subjects (3.5%, *P*<0.001) compared to their Canadian counterparts (2.5%, *P*<0.003).

**Table 5:** Population average for the 10-year predicted cardiovascular disease risk.

Population	Predicted 10-Year CVD risk (%)				*P*^*1*^		β Estimates of linear regression (%)^3^				
	HCV+		HCV-			Model 1^4^			Model 2^5^		
	*n*	Mean±SD	*n*	Mean±SD		β	95% CI	*P*	β	95% CI	*P*
**CHMS**	87	10.5**±**8.8	10028	8.0**±**6.6	0.008	2.47	0.65–4.30	0.008	2.49	0.83–4.16	0.003
**NHANES**	326	14.7**±**10.3	16342	10.0**±**10.0	<0.001	4.21	3.12–5.30	<0.001	3.48	2.48–4.48	<0.001
***P***^***2***^	<0.001		<0.001								

^**1**^Significance for the difference in CVD risk (estimated from by the FRS) between HCV infected and non-infected subjects (t-test).

^**2**^Significance for the difference in CVD risk (estimated from by the FRS) between participants from CHMS and NHANES within each HCV-infection subgroup (t-test).

^**3**^Linear regression models are for risk of CVD (estimated from by the FRS) in hepatitis C virus infected cases vs. non-infected controls.

^**4**^Unadjusted model.

^**5**^Multivariate model adjusted for ethnicity, number of metabolic syndrome components, and body mass index.

We did not control for CVD risk factors that were already included in the FRS algorithm.

## Discussion

Results of the present study suggest that HCV infection is associated with an increased 10-year risk of CVD and bolsters the findings of previous meta-analyses [6, 48] evaluating CVD risk and mortality in HCV-positive patients. Based on data from national surveys, sampled from the general population of Canada and the United States, our results suggest that 10-year CVD risk was 2.5–3.5% higher in HCV-infected subjects compared to the HCV-negative population. In the present study, the 10-year risk of CVD was estimated to be approximately 8% and 10% in the general Canadian and American populations, respectively (Table 5). Similar estimates were previously observed in both populations from the national representative surveys evaluated in our study. Using the same methods to calculate the FRS, Setayeshgar et al., reported the 10-year CVD risk in Canadian adults (from the CHMS study) to be 8.1% [42]. In the American general population, however, Lopez-Jimenez et al., assessed 10-year CVD risk from the NHANES survey to be 11% [49]. These estimates are similar to what we observed in the HCV-negative subjects.

In the HCV-infected subjects, the CVD risk estimates generated from the NHANES survey was 14.7% (Table 5). This risk level was comparable to the 10-year CVD risk of ~12% previously assessed in the same study population [50]. To our knowledge, the present study is the first to estimate the 10-year risk of CVD in HCV infected subjects and the association between the two conditions in the Canadian population using population-based survey data. Our results suggest an approximately 11% elevated CVD risk due to HCV infection in the Canadian population. In the Ontario HIV Treatment Network Cohort Study, the rates of CVD events in a large cohort of HIV mono-infected and HIV/HCV co-infected patients was previously evaluated [51]. In that study, the authors reported a 1.2-fold higher incidence of CVD events and a 1.4-fold elevated risk of CVD in the co-infected patients compared to those mono-infected with HIV [51].

We noted differences in the prevalence of HCV in relation to the marital status, age, schooling, income, the smoking status and the frequency of the intravenous drug use (Table 1). Similar sociodemographic characteristics were observed in different HCV infected populations around the world [52–55]. These findings may facilitate a better understand of the HCV transmission modes in North America and enable effective approaches for intervention. Furthermore, in our study population, a distinctive pattern of various cardiometabolic risk factors was observed in the HCV-infected patients (Table 2). Increasing severity of liver diseases has long been associated with progressively lower lipid levels and a range of metabolic disorders. For example, the induction of hepatic steatosis was found to be a risk factor leading to insulin sensitivity, metabolic abnormalities and visceral obesity [56]. Our results (Table 2) confirm previous reports demonstrating lower levels of LDL-C [57, 58], TGs [59] and ApoB [60] at the various stages of chronic HCV infection. This disrupted pattern of serum lipid profile can be the result of deteriorating liver functions [61] indicating that virus itself may be involved in the imbalance of serum lipoproteins/apolipoproteins [60] which is a known risk factor in atherosclerosis and CVD [62]. In support, several lines of recent evidence indicate that HCV by itself or factors associated with the infection can promote the occurrence and progression of atherosclerosis [63, 64]. For example, a recent meta-analysis indicates that HCV infection is significantly associated with carotid atherosclerosis, independent of classical risk factors such as type 2 diabetes, insulin resistance or hepatic steatosis [63]. HCV-positive participants from NHANES had lower cholesterol than their noninfected counterparts from the same survey, and further that HCV status is associated with lower BMI in both surveys. These findings can be explained by the recent reports showing that patients with HCV genotype 3 have lower total cholesterol than other infected patients with different genotypes [65]. Additional evidence for the role of viremia in CVD risk and its effect on cardiometabolic risk factors can be substantiated from our results (between the two surveys) demonstrating that in HCV cases who were also positive for the viral DNA there is lower total cholesterol, LDL-C and total cholesterol:HDL-C ratio than the RNA-negative HCV infected cases. Further, lower BMI can be linked to the status of persons who inject drugs of these patients. Moreover, elevation of proinflammatory cytokines in chronic HCV may be involved in the progression of atherosclerosis [9]. Although the levels of proinflammatory cytokines were not assessed in the two evaluated surveys, the downstream acute phase reactants (APR) such as CRP (NHANES and CHMS), fibrinogen (CHMS) and homocysteine (CHMS) were examined. In the present study, both CRP and fibrinogen levels were lower in the HCV-infected subjects compared to their non-infected counterparts (Table 2). In agreement, several studies have shown that CRP levels are downregulated in HCV patients [66–68] and plasma fibrinogen concentration progressively decreases as liver cirrhosis worsened [69] in patients with hepatic-nephrotic disease [70]. Taken together, it is possible that HCV infection (and/or replication) may blunt CRP synthesis [68] and lead to a state of acquired fibrinogen deficiency [69]. These interrelationships may however not explain the profiles of CRP and fibrinogen in the present study populations—as fibrosis parameters and platelet count (a good measure of chronic liver disease stage)—were not measured. A close relationship may be suggested between the severity of cirrhosis and alteration in the hemostatic regulation of the fibrinolytic system that is closely linked to control of inflammation and a role in CVD risk [9]. Indeed, activation of inflammatory processes, disruption of cytokine synthesis and imbalance in APR homeostasis are well-known risk factors in the increased CVD risk [48, 71, 72]. Overall, it was proposed that liver cirrhosis in HCV infection may accelerate atherosclerosis [48, 73, 74] leading to higher prevalence of carotid plaques [75] and increased risk of cardiovascular morbidity and mortality [76, 77]. Several factors were also proposed to mediate the link between HCV infection and risk of CVD [48, 78]. These include: augmented oxidative stress, modified iron homeostasis [48, 79], activation of immunological and/or inflammatory processes leading to a disrupted cytokine imbalance [48, 80] and induction of hepatic steatosis, a risk factor in insulin sensitivity and related metabolic abnormalities [56].

This study has several limitations which should be considered. For example, the limited data on intravenous drug use did not permit including this factor into our model. As expected, this behavior was highly associated with HCV infection as previously noted in the Canadian users [81]. Furthermore, HIV status was not captured into our study. The NHANES survey uses laboratory testing on 20- to 59-year-old participants whereas this was not available for the CHMS except for the self-reported information. This discrepancy rendered comparison between the two survey studies not feasible given the potential for bias of self-reported data inherent in conducting analyses on population-based surveys. HIV status has been linked to CVD risk in subjects co-infected with HCV [50]. Another limitation of the present study is that we were unable to examine the effect of ethnicity on the CVD risk in HCV patients. Ethnicity did not influence the CVD risk in the in the Canadian HCV positive patients (*P* = 0.41, ANOVA test). However, in the NHANES cohort, HCV-positive White subjects had a significantly higher (*P*<0.001) FRS score (18.0 **±** 10.5) than that in the other ethnic groups (African Americans: 14.2 **±** 10.6; Asians: 17.4 **±** 11.9; others 12.4 **±** 9.3). In both populations nevertheless, a suggestion of any ethnic-related CVD risk difference in HCV patients cannot be substantiated as it is severely underpowered by the small number of participants from the different ethnic groups. Moreover, our inability to combine the two population samples due to the different approaches taken in estimating various risk factors may have hindered acquiring a large sample size—particularly from the Canadian survey—to improve the outcome of our study. However, the definition of HCV status used was based on laboratory-confirmation after initial HCV screening was carried out using antibody tests. This protocol is known to result in significant reduction of false-positives and an accurate exposure definition [42, 49]. In this regard, there was no significant difference in CVD risk between HCV-RNA positive and negative status in those who had a positive anti-HCV result in participants from both the NHANES (P = 0.85) and CHMS (P = 0.21) surveys. This is critical as the direct-acting antiviral therapies offer sustained virologic response rates of almost 100%, the CVD risk may be decreased after successful antiviral therapy, a response that can be compared between cases with anti-HCV positive and HCV-RNA negative tests and those with anti-HCV positive and HCV-RNA positive test results. It was not surprising to find no difference in the FRS score between the two segregated groups mentioned above (i.e., anti-HCV positive/HCV-RNA positive cases vs. anti-HCV positive/HCV-RNA negative cases) given the small sample size of the RNA positive cases within the studied populations and also in light of the well-known large interindividual variation within the FRS factors that is usually present in humans. Overall, our results are in line with previous estimates of CVD risk from the same two populations [42, 49, 50]. Using data from two general population surveys produced two nearly similar estimates which represent the HCV-infected and -uninfected subpopulations of the US and Canada.

In conclusion, using data from two general population surveys from Canada and the USA, we report 11–15% elevated 10-year risk of CVD in HCV-infected individuals with no history of this chronic condition at baseline. When the relationship between HCV infection and CVD is adjusted for confounders there was 2.5–3.5% absolute increased risk of cardiovascular events due to HCV infection. Using cross-sectional surveys in the present study resulted in similar estimates of the 10-year risk of CVD when compared to prospective studies, e.g., in the Canadian population [51]. Patients with HCV may, therefore, benefit from a frequent monitoring of surrogate markers of CVD risk such as the range of cardiometabolic factors and inflammatory markers. This may assist in establishing more specific CVD prevention strategies. Although studies from the US [82] and Canada [83] indicate a shift in HCV seroprevalence and decreased infection rates in younger generations, the burden of the disease on the healthcare cost remains significant. For example, in Canada, it has been estimated that chronic HCV costs are projected to rise by 60% by 2035 [84]. The recent availability of large-scale HCV therapy using direct-acting antivirals that is both highly effective and well-tolerated may aid in reducing the projected healthcare cost associated with HCV infection. Furthermore, apparently viremia is an important factor in the risk prediction of future CVD. This can be demonstrated from the significantly lower 10-year disease risk in HCV-positive cases (from the NHANES population) who were also positive for the viral RNA. Indeed, similar results were not attainable from the Canadian cases (due to the small size) and this outcome was generated from a limited number of US cases. However, this increased 10-year CVD risk in viremia may substantiate its critical role in the development of the chronic disease and—more importantly -that antiviral therapy should be highly encouraged as it can directly improve the cardiovascular risk in addition to its effect on the infection outcome. Understanding the relationship between HCV and CVD, and how this relationship is affected by population-level HCV reductions, has public health implications for CVD control, a leading cause of morbidity worldwide.

## Supporting information

S1 Table Number of hepatitis C virus (HCV) infected patients stratified by the method of HCV detection. (PDF)
