# Supplementary material for: Cardiovascular disease risk in patients with hepatitis C infection: Results from two general population health surveys in Canada and the United States (2007-2017)
Source: PLoS One. 2018 Dec 12;13(12):e0208839. doi: 10.1371/journal.pone.0208839 (PMC6291240; doi:10.1371/journal.pone.0208839)
Supplement: S1 Table — (PDF) [file pone.0208839.s001.pdf]

Cardiovascular disease risk in patients with hepatitis C infection: Results from two general population health surveys in Canada and the United States (2007-2017)

Alaa Badawi, Giancarlo Di Giuseppe, Paul Arora

Supplementary Materials

S1 Table. Number of hepatitis C virus (HCV) infected patients stratified by the method of HCV detection*.

Study population	Antibody Testing	RNA Testing		
		**Positive (%)**	**Negative (%)**	**Not done (%)**
NHANES (n=326)	Positive	167 (51.2)	48 (14.7)	22 (6.8)
	Negative			
	Indeterminant	2 (0.6)		
	Not done	87 (26.7)		
CHMS (n=87)	Positive	14 (16.1)	7 (8.0)	66 (75.9)
	Negative			
	Indeterminant			
	Not done			

*Empty cells represent no sample in this category.
